# Case Report: A case of radioactive iodine-refractory thyroid cancer accompanying cervical lymph node metastasis treated with US-guided RFA combined with ^125^I seed implantation

**DOI:** 10.3389/fonc.2022.987484

**Published:** 2022-12-01

**Authors:** Yuanpeng Zhai, Yu Shao, Qian Li

**Affiliations:** Department of Ultrasound, The Affiliated Cancer Hospital of Zhengzhou University & Henan Cancer Hospital, Zhengzhou, China

**Keywords:** ultrasound- (US) guided, radiofrequency ablation (RFA), ^125^I seed implantation, radioactive iodine-refractory (RAIR) thyroid cancer, cervical lymph node metastasis

## Abstract

**Background:**

Local control of metastases is critical to improving the life quality of patients with radioactive iodine-refractory (RAIR) thyroid cancer accompanying regional lymph node metastasis.

**Case report:**

The reported patient suffered from RAIR thyroid cancer accompanying poorly controlled cervical lymph node metastasis. The patient’s lesions were controlled through ^125^I seed implantation combined with ultrasound-guided radio-frequency ablation (US-guided RFA). Such a combination therapy has not been reported to date.

**Conclusion:**

This study found US-guided RFA combined with ^125^I seed implantation to be safe and effective for the control of cervical local metastases in patients with RAIR thyroid cancer.

## Background

In recent years, the incidence of thyroid cancer in China has been increasing, with differentiated thyroid cancer (DTC) a dominant type. Some patients with DTC can develop radioactive iodine-refractory differentiated thyroid cancer (RAIR-DTC) as they gradually lose the capacity to uptake iodine during disease treatment and progression (1). Patients with RAIR-DTC accompanying regional lymph node metastasis struggle with poor prognosis because they cannot benefit from ^131^I diagnosis and treatment. Although conventional diagnosis and treatment methods employed in clinical practices, such as targeted medical therapy and surgery, can improve the prognosis of patients with RAIR-DTC to a certain extent, the overall response is not satisfactory. Some patients suffer from skin rupture caused by poorly controlled metastases, which seriously affects their quality of life. Studies have shown that ^125^I seed implantation in the treatment of RAIR-DTC accompanying regional lymph node metastasis can safely deliver a good local control effect, making it of clinical value (2). However, the effectiveness of this treatment is usually limited to metastases of a small size. When lymph node metastases are large, with skin rupture and internal liquefactive necrosis, ^125^I seed implantation alone has limitations. For example, in metastases of this kind, it is not easy to control the location of the implanted seeds since they are likely to fall into the liquid components, thereby reducing the therapeutic effect. New and effective methods are needed for controlling metastasis locally. As a minimally invasive treatment method, RFA has been used to treat benign thyroid nodules, papillary thyroid cancer and recurrent thyroid cancer (3–6). It is safe and feasible, especially when patients refuse surgery, have high requirements for aesthetics or have surgical contraindications, RFA as an alternative treatment can reduce patients’ anxiety and improve their quality of life (7). In our case,during the operation the patients was under monitoring and questioning, the operation was sucessfull and safe.

This report presents the results achieved using ultrasound- (US) guided aspiration of metastatic liquid components, radio-frequency ablation (RFA), and ^125^I seed implantation in the local control of metastases.

## Case report

A 70-year-old woman was admitted to our hospital with “lung metastasis after papillary thyroid carcinoma surgery and new cervical masses detected over 2 years.” Two years earlier, in May 2004, she had received a thyroid mass resection under local anesthesia in a local hospital to remove the cervical mass; subsequently, the postoperative pathology revealed lymph node metastasis of papillary thyroid carcinoma. With a recurrent cervical mass, the patient visited our hospital for the first time in August 2006 and underwent a radical resection of a left thyroid carcinoma under general anesthesia. The postoperative pathology indicated papillary thyroid carcinoma. The patient continued to suffer regional lymph node metastasis and received lymph node dissection twice in combination with ^131^I therapy.

### Operation history

In October 2010, the patient received a left neck dissection under general anesthesia in our hospital. Postoperative pathology indicated that one in five level-III and -IV lymph nodes had metastases of papillary thyroid carcinoma, with level VI showing the formation of a cancerous node. On March 26, 2015, the patient received a radical resection of recurrent thyroid cancer and a right recurrent laryngeal nerve anatomic exploration. The postoperative pathology indicated the infiltration of papillary thyroid carcinoma at levels IV and VI of the left neck. At levels IV and VI of the right neck, all the four lymph nodes detected were found with metastases of papillary thyroid carcinoma, accompanied by the formation of a cancerous node.

### History of ^131^iodine therapy

The patient received ^131^I treatment in our hospital in December 2011, May and October 2012, May and October 2013, August 2014, June 2015, April 2016, and April 2017, with doses of 150, 150, 150, 100, 150, 150, 150, and 100 mCi, respectively.

According to the patient’s latest physical examination, she had multiple palpable enlarged lymph nodes in the neck, with tough texture and poor mobility. In 2014, new masses were detected in the right neck, which eventually ruptured in 2019. The patient received a lymph node puncture in the right neck on November 30, 2019, in the Nuclear Medicine Department of the Shanghai Sixth People’s Hospital (affiliated with Shanghai Jiao Tong University). An analysis showed papillary thyroid carcinoma due to the BRAF V600E mutation. The ruptured lesion in the right neck was not under control, and a new mass was found in the left neck, protruding through the skin. This mass had a diameter of around 30 mm and was accompanied by bruising of the local skin. The patient’s quality of life was significantly affected. The patient was not provided with a therapeutic regimen after visiting upper-level hospitals several times, so she returned to our hospital. After admission, the possibility of lymph node metastasis was considered based on a contrast-enhanced computed tomography (CT) scan of the neck showing multiple nodules and tumor shadows in the thyroid region, bilateral parotid region, bilateral neck, bilateral subclavian and supraclavicular fossae, and the mediastinum. Thyroglobulin in the puncture fluid from the right cervical ruptured lesion was measured at 413 ng/ml, and the lesion was considered to be a thyroid lymph node metastasis. In recent years, the patient had undergone several surgeries, ^131^radioiodine therapy, systemic anti-tumor therapies, and treatment with sorafenib, vemurafenib, and pembrolizumab. Unfortunately, the effects were not satisfactory, and the metastases in the cervical lymph nodes remained uncontrolled. At that point, the patient’s condition was severe, and there was no effective anti-tumor therapy.

After admission, the patient was given targeted systemic anti-tumor treatment comprising anlotinib (1 tablet/day) and Euthyrox (112.5 µg/day) as inhibition and replacement therapy. As the left cervical mass was relatively large, local cytoreductive surgery was considered for lowering the risk of skin ulceration. Given the large volume of the left cervical mass, with a diameter of approximately 30 mm, a total of 33 ^125^I seeds were implanted one by one at an interval of 5 mm under CT scan guidance to achieve local cytoreduction for the metastases and reduce the risk of skin rupture.

**Figure 1:**
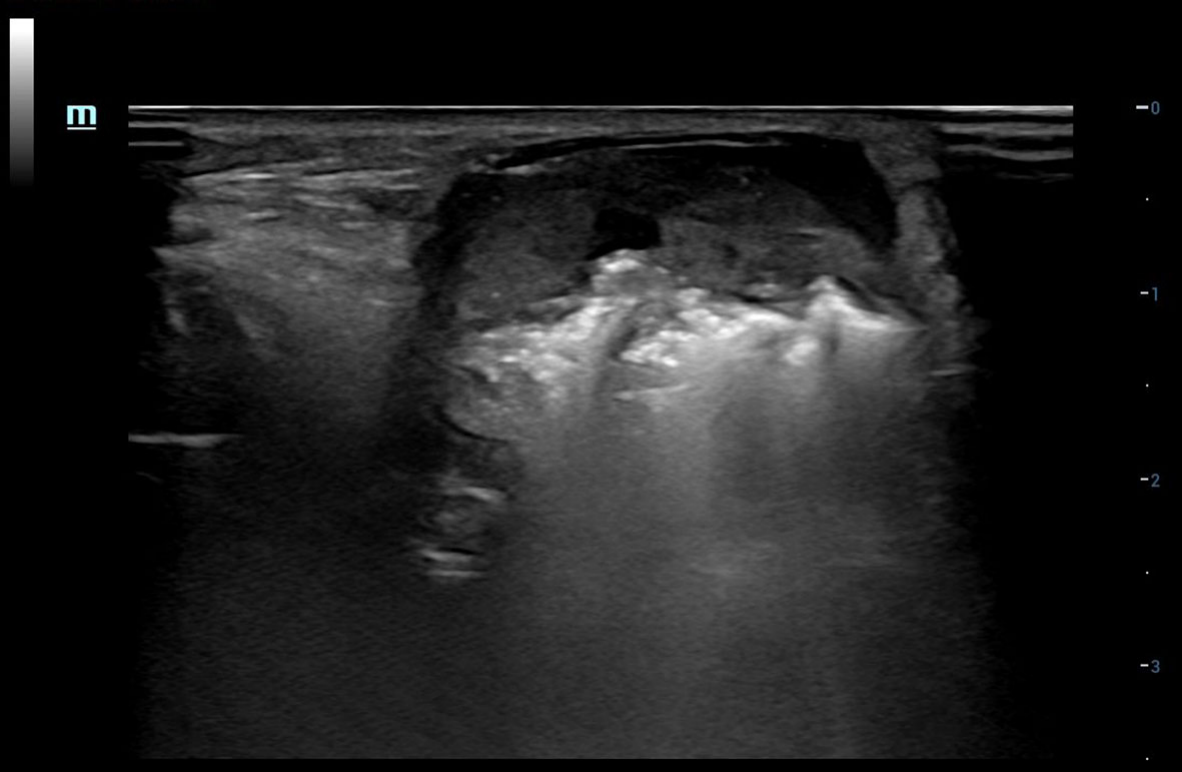
US-guided RFA for left cervical mass.

**Figure 2:**
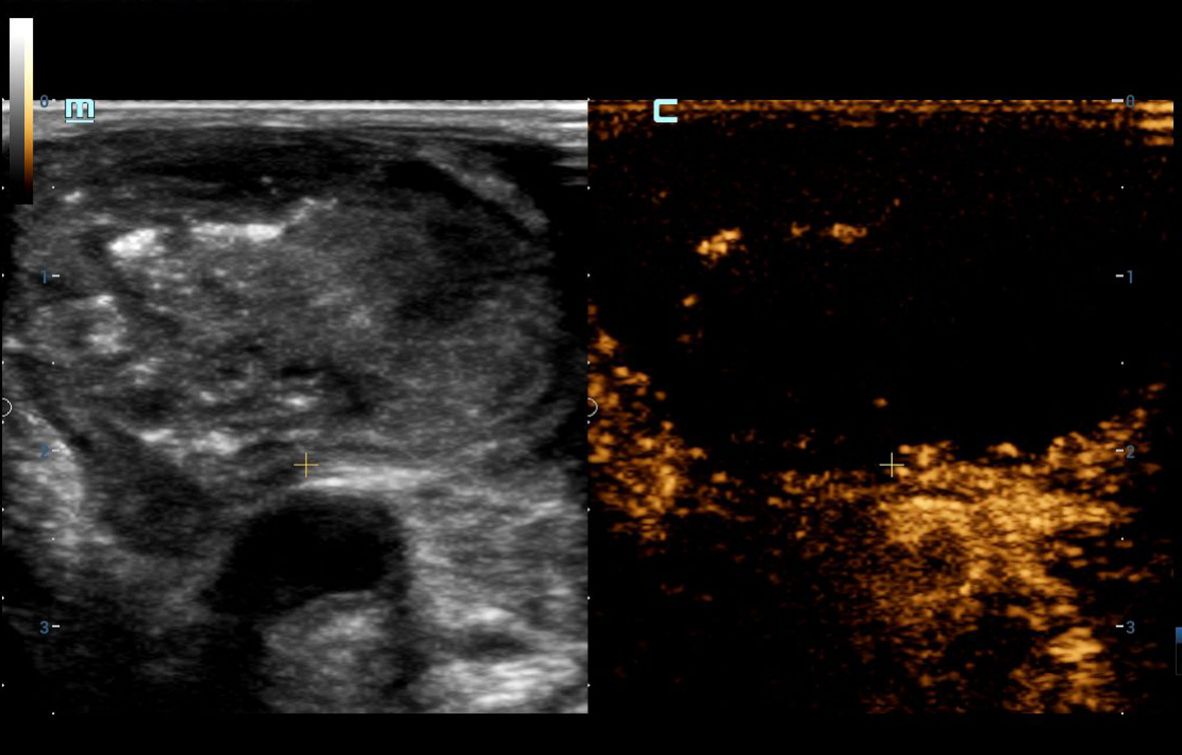
No contrast enhancement in left cervical mass according to postoperative ultrasonography.

Three days after ^125^I seed implantation in the left cervical metastases, the patient reported feeling slightly better. However, the mass in her left neck remained large; it was accompanied by liquefactive necrosis, and seeds were entering the liquid components. To prevent further enlargement and development of the metastases, as well as to avoid radiation damage to adjacent tissues and organs, we proposed during multidisciplinary consultations that RFA be used to reduce the metastases in combination with other therapies for symptomatic treatment. Under the guidance of US, the size of the left cervical mass was measured to be 33 × 19 mm, which protruded through the surface of the skin. Accordingly, we aspirated the liquid components in the lesion, considering the continuous new production of fluid, seed implantation was not carried out, but RFA inactivation was performed on the solid portion at the same time as fluid extraction (**Figure 1**). Postoperative ultrasonography showed no obvious contrast enhancement in the metastatic lymph nodes of the left neck (**Figure 2**). The left lesion recover over the month following the RFA. After 23 days, the metastases no longer protruded through the skin, and all that could be seen on the cervical skin was a slight crust. Thirty days after the RFA, a contrast-enhanced CT scan of the neck revealed that the cervical anterior subcutaneous nodule could no longer be seen, which was a postoperative change. The patient’s left neck completely recovered 32–35 days after the RFA.

The findings of the contrast-enhanced CT scan and the recovery of the patient’s left neck provide evidence that US-guided RFA combined with ^125^I seed implantation can deliver a complete local response for large metastases in a safe, effective manner. US-guided RFA is particularly effective in treating lymph node lesions with fluid.

**Figure 3:**
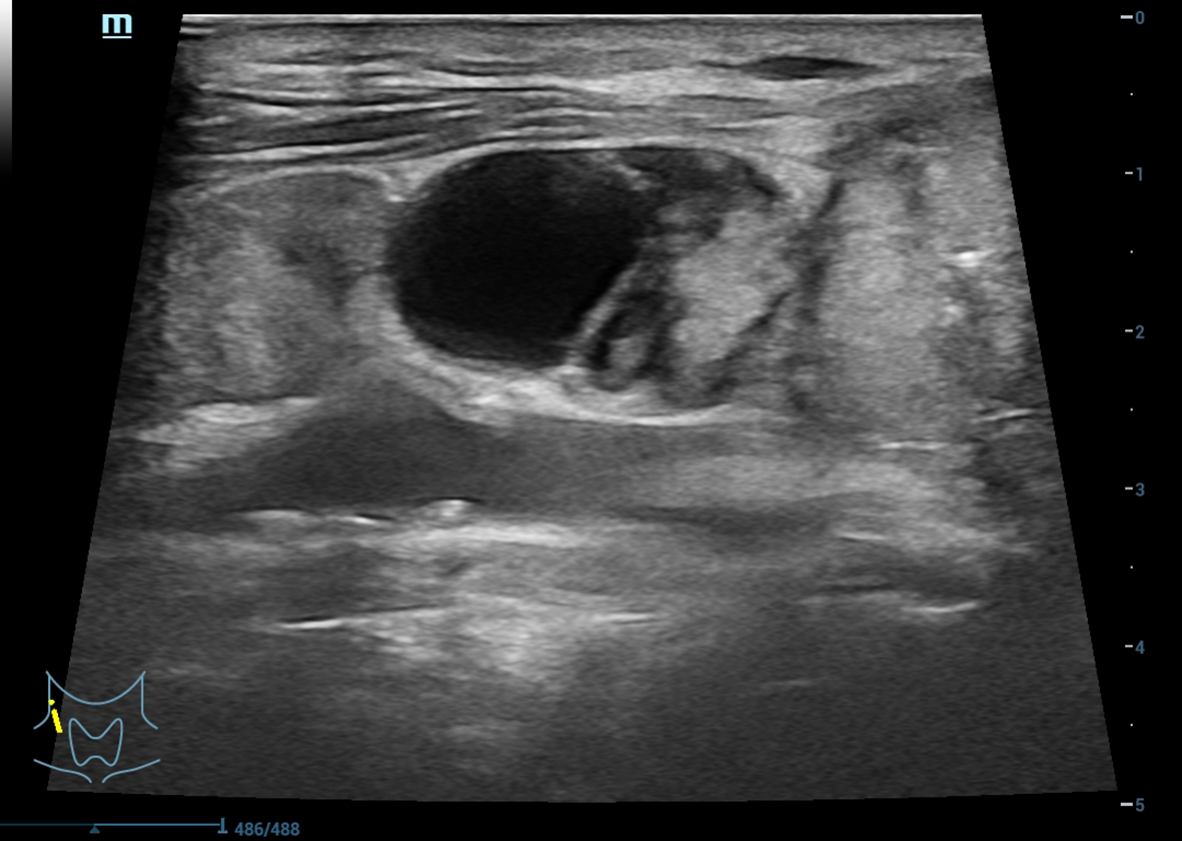
Metastatic lymph nodes accompanying internal liquefactive necrosis in right neck as shown in ultrasonography.

**Figure 4:**
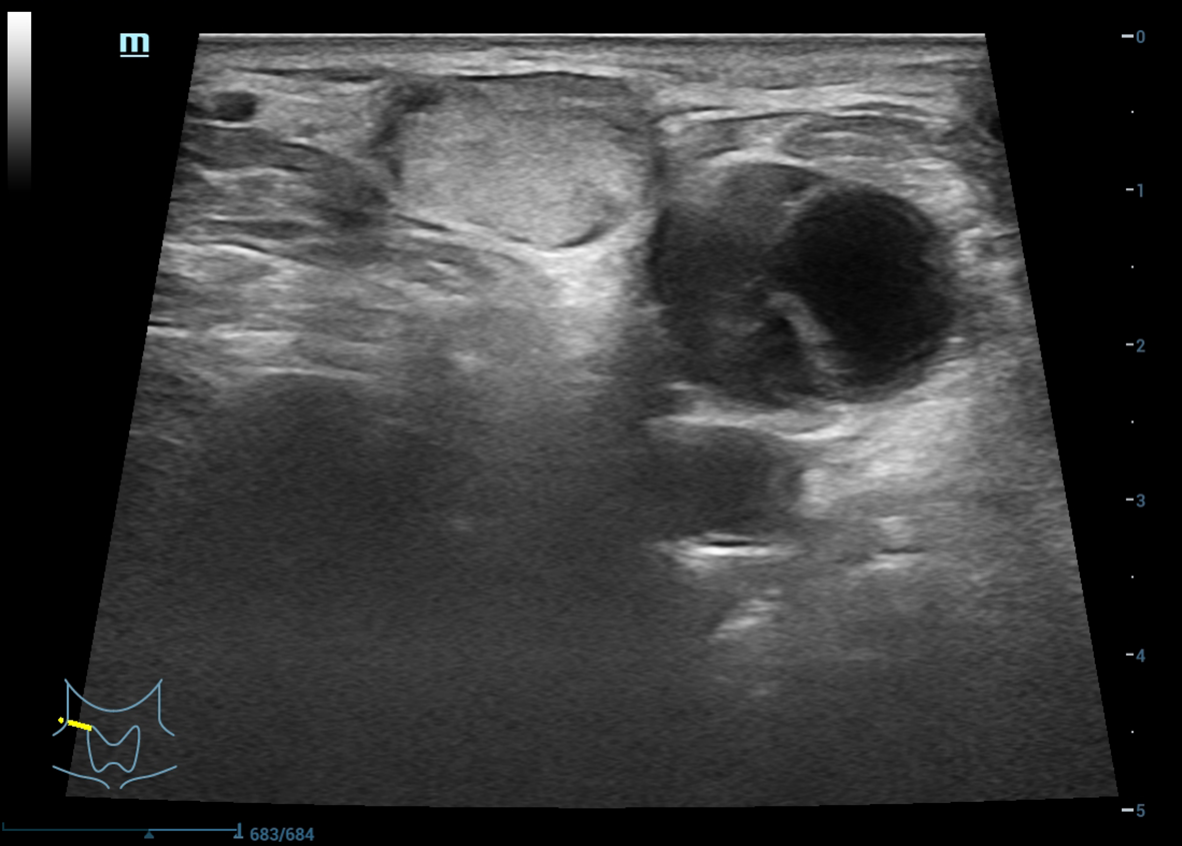
Right cervical lymph node metastasis.

As the therapeutic effect of US-guided RFA for the patient’s left cervical metastases was quite considerable, we continued with RFA of a fluid-containing lymph node metastasis in the patient’s right neck at a safe location. The US-guided measurements showed one of the lymph nodes in the right neck was around 21 × 11 mm, another was around 24 × 17 mm (**Figures 3**, **4**), and there were heterogeneous internal echoes.

We aspirated the liquid components and performed RFA. According to postoperative ultrasonography, no obvious contrast enhancement was found in the cervical metastatic lymph nodes, and there was no residual ablation. The day following the right cervical lymph node RFA, there was a special location of one ulcerated lymph node on the right neck, and there was no safe entry point, so seeds were implanted in the patient’s right cervical ruptured lymph node. The ruptured mass shrank following the implantation of nine ^125^ I one by one at an interval of 5 mm.

Two months later, the mass had gone. The ablated cervical mass disappeared over 40 days. Following this success, the masses of the patients in the subsequent treatment were all solid masses, and the location was deep adjacent to the important cervical nerve, so the patient had three more occasions when ^125^I seeds were implanted into the cervical lymph nodes: 9, 16, and 9 seeds were implanted, respectively. Now, the patient is recovering well, with the local metastases in the cervical lymph nodes well under control. She was discharged from hospital and is receiving systemic targeted drug therapy.

## Discussion

Most patients with DTC achieve a good prognosis after receiving surgery, ^131^I radiotherapy, thyrotropin inhibition, and other therapies, with a 10-year survival rate of up to 90%. However, about 15% of these patients develop local recurrence and/or distant metastases and have a 10-year survival rate below 10% (8). As some cases are not sensitive to radioactive iodine therapy, they may develop RAIR-DTC, which is characterized by fast progression and high mortality. It is difficult for these patients to achieve satisfactory results with the traditional methods referred to above.

Currently, the diagnosis and treatment of RAIR-DTC are controversial. According to the usual definition of RAIR-DTC, in the absence of exogenous iodine load interference when the level of thyroid-stimulating hormone is greater than 30 m IU/L, the lesion loses its iodine uptake capacity. Consequently, favorable results are not expected from ^131^I treatment. In the case of the present study, the patient’s condition was severe. Her cervical metastases could not be surgically removed, and a high dose of radioactive iodine, if allowed to accumulate around her neck, would likely have caused radiation damage to the larynx and tracheal mucosa, resulting in radiation tracheitis or radiation pneumonia. In this case, the patient was treated as RAIR-DTC because radioactive iodine therapy was not feasible for her (9). The treatment for RAIR-DTC is controversial, and cases accompanying cervical regional lymph node metastasis remain a problem to be solved (10). Surgery, chemotherapy, targeted therapy, and other related methods are now applied in clinical practices. In this case, the patient’s condition was severe, and her cervical metastases could not be surgically removed. As studies in molecular biology on thyroid cancer have advanced, a large number of targeted therapeutic drugs have been developed in recent years (11–13), making targeted drug therapy a major option for patients with RAIR-DTC. However, this patient did not respond well to sorafenib, vemurafenib, pembrolizumab, and other targeted therapy drugs. Chemotherapy is mainly used for terminally ill patients with obvious invasive symptoms which are uncontrollable *via* radioactive iodine therapy or surgery. However, as most patients with RAIR-DTC are not sensitive to chemotherapy, traditional chemotherapy drugs are unsuitable (14).

For 15 years (2004–2019), the patient suffered from recurrent cervical lymph node metastases, which progressively increased in size and number and were not effectively controlled. Patients need an effective local therapy that includes surgery and external irradiation, with a focus on cytoreduction, to improve the quality of life (15). However, in this case, the patient’s condition was severe, and the cervical metastases could not be surgically removed. The patient was not provided with any effective therapeutic regimen despite repeatedly visiting several hospitals. After the patient came to our hospital, we initially treated the cervical metastases with ^125^I seed implantation and US-guided RFA.

The lymph node metastases in her left neck, which were large with liquefactive necrosis, showed a positive therapeutic effect in the early stage of ^125^I seed implantation. Due to a short radiation distance, the ^125^I seeds can effectively kill tumor cells in lesions with little damage to surrounding tissues, thus achieving highly conformal brachytherapy (2). Nonetheless, its effect on killing cancer cells depends on precise positioning and reasonable arrangement. Studies have shown that the larger the lesion, the lower the therapeutic effect. This has been attributed to seed displacement due to liquefactive necrosis in the large lesions, which makes it impossible to precisely position and reasonably arrange the seeds. This leads to a lower conformal index of dose distribution in the lesion, thereby weakening the therapeutic effect. Furthermore, dose deposit by seed displacement poses a greater risk of radiation damage to healthy tissues, making inflammatory reactions more likely, forming adhesions between lesions and vital organs (such as peripheral vessels), and thus making further treatment, including replantation, more difficult (16). In this case, the patient had large lymph node metastases accompanied by liquefactive necrosis, and the US showed that implanted seeds had migrated into the liquid components. Considering the unsatisfactory nature of iodine seed implantation, it is important to explore new therapies for controlling local lesions.

During multidisciplinary consultations, our department suggested that the liquid components of metastasis be treated with US-guided aspiration and metastasis be treated with RFA. The RAF can be used for patients with recurrent or metastatic lymph nodes after standard surgical resection and neck lyphnode dissection (17). The patient had undergone at least one operation before, the anatomical structure of the operation area has changed and adhesions would be severe. High risk and difficulty hindered the second operation. besides, the patient is not easy to accept the second operation. In addition, some patients may be ineffective to the iodine therapy, these patients requires further local surgery or RFA. RFA has the unique advantages of safety and simple operation, which provides a more reliable treatment option (7).

The RFA technique has been increasingly applied in clinical practice since it is minimally invasive, has a beneficial therapeutic effect, and is well tolerated. The RFA needle is inserted into lesions under the guidance of US, inducing a thermal effect *via* a high-frequency alternating current in the targeted tissues, thereby inactivating lesion cells.

In this case, the left cervical lymph node metastasis was large (33mm×19mm) and accompanied with liquefaction necrosis. 125I was not effective at first for the particles fell into the liquid components. Under the guidance of ultrasound, we aspirated the liquid components of the left cervical lesion. Considering that the liquid would be generated continuely, we did not implant the particles again, but chose to inactivate the solid parts while aspirating the liquid. RFA was then performed on metastatic tumors at safety site of the right cervical. The solid mass was deep adjacent to important cervical nerves, so 125I was selected for the following treatment.

After RFA, the metastases in the patient’s left and right neck shrank. The metastases that ruptured in the upper part also shrank after the implantation of ^125^I seeds. In summary, the patient obtained the beneficial effect of local control of the metastases through US-guided RFA combined with ^125^I seed implantation. This is a safe method with high clinical application value.

In this case, RFA plays an important role in the safe location of fluid lymph node lesions, and seed implantation plays an important role in the subsequent treatment of lymph node lesions located deep adjacent to important cervical nerves in the right neck. The implantation of 125I seeds has good local control effect, high safety and high clinical application value for the patients with RAIR-DTC with regional lymph node metastasis. Especially, when the tumor showed liquid necrosis, the implantation position of 125I particles would be uncontrolled. If the particles fall off into the liquid components, the effectiveness will be greatly reduced. RFA has been used to treat benign thyroid nodules, papillary thyroid cancer, recurrent thyroid cancer and lymph node metastasis safely. The coagulative necrotic tissue gradually becomes smaller after the RFA operation, and the clinical symptoms caused by nodules are also significantly improved, which is safe and feasible. Besides, the solid part can be inactivated while the fluid is pumped for mixed lesions.

## Conclusions

In conclusion, patients with RAIR-DTC with accompanying cervical lymph node metastases face limited therapy options and a poor prognosis. In addition to conventional therapies, such as surgery, chemotherapy, and targeted drug treatment, new local therapies are needed, particularly for lesions with large local mass accompanied by liquefactive necrosis. For such lesions, US-guided RFA is a valuable contribution to these therapies. Our patient was treated with targeted drugs and thyrotropin inhibition, combined with US-guided RFA and ^125^I seed implantation for local control, to achieve a complete clinical response for RAIR-DTC with accompanying cervical lymph node metastases.

## Data availability statement

The raw data supporting the conclusions of this article will be made available by the authors, without undue reservation.

## Ethics statement

This study was conducted with approval from the Ethics Committee of the Affiliated Cancer Hospital of Zhengzhou University & Henan Cancer Hospital. The patients/participants provided their written informed consent to participate in this study.

## Author contributions

Conception and design of the research: YZ, YS. Acquisition of data: QL. Analysis and interpretation of the data: YZ. Statistical analysis: YZ, YS. Obtaining financing: YZ. Writing of the manuscript: YZ, Critical revision of the manuscript for intellectual content: QL. All authors read and approved the final draft.

## Funding

This study was funded by the Scientific and technological project in Henan Province (No.212102310637).

## Conflict of interest

The authors declare that the research was conducted in the absence of any commercial or financial relationships that could be construed as a potential conflict of interest.

## Publisher’s note

All claims expressed in this article are solely those of the authors and do not necessarily represent those of their affiliated organizations, or those of the publisher, the editors and the reviewers. Any product that may be evaluated in this article, or claim that may be made by its manufacturer, is not guaranteed or endorsed by the publisher.

## References

[1] Schmidt A Iglesias L Klain M Pitoia F Schlumberger MJ . Raioactive iodine –refractory differentiated thyroid cancer: An ucommon but challenging situation. Arch Endocrinol Metab (2017) 61:81. doi: 10.1590/2359-3997000000245 28225999 PMC10522117

[2] Ye SF Zhou YX Shou F Zhang JJ Chen Y . Clinical study on clinical study on 125I seeds implantation for colon cancer patients with liver I seeds implantation for colon cancer patients with liver metastasis. Oncol Prog (2018) 16:609 –612. doi: 10.11877/j.issn.1672-1535.2018.16.05.22

[3] Teng D Sui G Liu C Wang Y Xia Y Wang H . Long-term efficacy of ultrasound-guided low power microwave ablation for the treatment of primary papillary thyroid microcarcinoma: A 3-year follow-up study. J Cancer Res Clin Oncol (2018) 144(4):771–9. doi: 10.1007/s00432-018-2607-7 PMC11813516 29427209

[4] Zhang M Luo Y Zhang Y Tang J . Efficacy and safety of ultrasound-guided radiofrequency ablation for treating low-risk papillary thyroid microcarcinoma: A prospective study. Thyroid (2016) 26(11):1581–7. doi: 10.1089/thy.2015.0471 27445090

[5] Kim JH Baek JH Lim HK Ahn HS Baek SM Choi YJ . 2017 Thyroid radiofrequency ablation guideline: Korean society of thyroid radiology. Korean J Radiol (2018) 19(4):632–55. doi: 10.3348/kjr.2018.19.4.632 PMC6005940 29962870

[6] Lee GM You JY Kim HY Chai YJ Kim HK Dionigi G . Successful radiofrequency ablation strategies for benign thyroid nodules. Endocrine (2019) 64(2):316–21. doi: 10.1007/s12020-018-1829-4 30569260

[7] Jeon YW Gwak HG Lim ST Schneider J Suh YJ . Long-term prognosis of unilateral and multifocal papillary thyroid microcarcinoma after unilateral lobectomy versus total thyroidectomy. Ann Surg Oncol (2019) 26(9):2952–8. doi: 10.1245/s10434-019-07482-w 31264119

[8] Liu YQ Lin YS . [Diagnosis, treatment and prognosis of differentiated thyroid carcinoma with iodine refractory]. Chin J Pract Surg (2019) 39:216–20. doi: 10.3390/ijms18061292

[9] Riesco-Eizaguirre G Galofré JC Grande E Zafón Llopis C Ramón y Cajal Asensio T Navarro González E . Spanish Consensus for the management of patients with advanced radioactive iodine refractory differentiated thyroid cancer. Endocrinol Y Nutricion Organo la Sociedad Espanola Endocrinol Y Nutricion (2016) 63:e17–24. doi: 10.1016/j.endonu.2015.08.007 26601805

[10] Bray F Ferlay J Soerjomataram I Siegel RL Torre LA Jemal A . Global cancer statistics 2018: GLOBOCAN estimates of incidence and mortality worldwide for 36 cancers in 185 countries. CA Cancer J Clin (2018) 68:394–424. doi: 10.3322/caac.21492 30207593

[11] Brose MS Nutting CM Jarzab B Elisei R Siena S Bastholt L . Sorafenib in radioactive iodine-refractory, locally advanced or metastatic differentiated thyroid cancer: A randomised, double-blind, phase 3 trial. Lancet (2014) 384:319–28. doi: 10.1016/S0140-6736(14)60421-9 PMC4366116 24768112

[12] Tahara M Schlumberger M Elisei R Habra MA Kiyota N Paschke R . Exploratory analysis of biomarkers associated with clinical outcomes from the study of lenvatinib in differentiated cancer of the thyroid. Euro J Cancer (2017) 75:213. doi: 10.1016/j.ejca.2017.01.013 28237867

[13] Brose MS Cabanillas ME Cohen EE Wirth LJ Riehl T Yue H . Vemurafenib in patients with BRAFV600E-positive metastatic or unresectable papillary thyroid cancer refractory to radioactive iodine: A non-randomised, multicentre, open-label, phase 2 trial. Lancet Oncol (2016) 17:1272–82. doi: 10.1016/S1470-2045(16)30166-8 PMC5532535 27460442

[14] Argiris A Agarwala SS Karamouzis MV Burmeister LA Carty SE . A phase II trial of doxorubicin and interferon alpha 2b in advanced, non-medullary thyroid cancer. Invest New Drug (2008) 26:183–8. doi: 10.1007/s10637-007-9091-2 17909728

[15] Jarzab B Dedecjus M Handkiewicz-Junak D Lange D Lewiński A Nasierowska-Guttmejer A . Diagnostics and treatment of thyroid carcinoma. Endokrynol Pol (2016) 67(1):74–107. 10.5603/EP.2016.0011 26884119

[16] Zeng XX Zhang WW Ji LQ Li G Wang YG Hao SS . Efficacy and safety of radioiodine-refractory differentiated thyroid cancer with regional lymph node or distant metastasis treated with 125I seed implantation. Xian Dai Zhong Liu Yi Xue (2020) 28:4256–61. doi: 10.3969/j.issn.1672-4992.2020.24.009

